# Shaving Bridges and Tuning Kitaraa: The Effect of Language Switching on Semantic Processing

**DOI:** 10.3389/fpsyg.2017.01438

**Published:** 2017-08-29

**Authors:** Suzanne C. A. Hut, Alina Leminen

**Affiliations:** ^1^Cognitive Brain Research Unit, Department of Psychology and Logopedics, Faculty of Medicine, University of Helsinki Helsinki, Finland; ^2^Institute of Behavioural Sciences, University of Helsinki Helsinki, Finland; ^3^Department of Clinical Medicine, Center of Functionally Integrative Neuroscience, Aarhus University Aarhus, Denmark

**Keywords:** semantics, language switching, second-language learners, N400, semantic processing, LPC, proficiency, ERP

## Abstract

Language switching has been repeatedly found to be costly. Yet, there are reasons to believe that switches in language might benefit language comprehension in some groups of people, such as less proficient language learners. This study therefore investigated the interplay between language switching and semantic processing in groups with varying language proficiency. EEG was recorded while L2 learners of English with intermediate and high proficiency levels read semantically congruent or incongruent sentences in L2. Translations of congruent and incongruent target words were additionally presented in L1 to create intrasentential language switches. A control group of English native speakers was tested in order to compare responses to non-switched stimuli with those of L2 learners. An omnibus ANOVA including all groups revealed larger N400 responses for non-switched incongruent stimuli compared to congruent stimuli. Additionally, despite switches to L1 at target word position, semantic N400 responses were still elicited in both L2 learner groups. Further switching effects were reflected by an N400-like effect and a late positivity complex, pointing to possible parsing efforts after language switches. Our results therefore show that although language switches are associated with increased mental effort, switches may not necessarily be costly on the semantic level. This finding contributes to the ongoing discussion on language inhibition processes, and shows that, in these intermediate and high proficient L2 learners, semantic processes look similar to those of native speakers of English.

## Introduction

A current estimate tells us that about 50% of the entire world’s population is bilingual (Grosjean, 2010). Even many more are still trying to master a second or third language. The dramatically increased need for learning multiple languages is particularly due to the growing importance of internet and online communication, and increasingly global work environments (Genesee, 2008). In the daily life of a bilingual speaker, it may be quite common to alternate between languages, for example by substituting a word in another language, or by mixing parts of a sentence altogether. Language switching has raised particular interest among bilingualism researchers, due to associated cognitive processes that inform us about the way multiple languages are processed and controlled.

Previous studies on language switching have often focused on the processing costs paired with switching from one language to another. These switch costs can be reflected as extra time needed to produce or recognise a word (e.g., Thomas and Allport, 2000; Costa and Santesteban, 2004). Switching effects have also been reported in neuro-imaging studies, such as increased N400 (e.g., Moreno et al., 2002) or N250 event-related potential (ERP) responses (e.g., Chauncey et al., 2008). Additionally, a switch in language seems to recruit neural regions related to executive control and domain-general inhibition (Abutalebi and Green, 2008; De Bruin et al., 2014).

Language switch costs are likely reflected by underlying language control processes, which are especially evident in cases where language dominance is involved. Language dominance is seen in many bilinguals who did not acquire their languages simultaneously, are not equally proficient in both languages, or have not received equal amounts of language exposure in their languages. Due to the language non-specific nature of bilingual lexical access (Dijkstra and Van Heuven, 2002; Marian and Spivey, 2003; Thierry and Wu, 2007), bilinguals are presented with unwanted interference from their other language(s), even if attempting to use only one of their languages at the time. This underscores the need for a language control system that reduces such interference and assures that items in a certain language can still be produced despite ongoing competition from the non-target language (e.g., Schulpen et al., 2003; Gollan et al., 2011).

The Inhibitory Control Model (IC Model, Green, 1998) views this control mechanism as the activation or inhibition of language schemas that belong to the languages in use and their lexical items (lemmas). Through activation of the appropriate language schema, lemmas that carry language tags belonging to the non-target language are inhibited. A neurocognitive adaptation of this IC model was later published by Abutalebi and Green (2008) to offer a valid neurobiological basis for this theory, which attributes language control to a network of frontal, parietal and subcortical regions. Later, the adaptive control hypothesis focused more strongly on the adaptivity of language control during the use of multiple languages, by specifying various control processes and how these in alter in different conversational settings (Green and Abutalebi, 2013). Recently, this control network has been reinforced with new evidence from neuroimaging studies (Abutalebi and Green, 2016).

Switch costs can be viewed as a reflection of the inhibitory processes that are at play during language selection. Furthermore, asymmetry in switch costs is suggested to be due to differences in language dominance. Switching to a more dominant language is more costly, because the baseline level of activation for the dominant language is high and therefore needs more suppression when the non-target language is used. In contrast, when using the dominant language, less active inhibition is needed to suppress the representation of the non-dominant language, as the baseline activation levels are assumed to be lower (Green, 1998). The IC Model is most frequently used to explain switch costs in production, but the Bilingual Interactive Activation (BIA; Dijkstra and Van Heuven, 2002) model proposes a similar inhibition mechanism for language reception. However, the BIA model suggests that inhibition takes place through inhibition of language nodes, and therefore places the locus of the control mechanism at the level of the lexicon, instead of the general executive system. Later, the BIA+ was developed to account for non-selectivity of language access and interlingual priming, and suggests that a higher-level task decision system monitors several task schemas and switches between them where necessary (Van Heuven and Dijkstra, 2010).

Language dominance has been found to have a profound impact on underlying language switching mechanisms. Switching to one language or another may prove difficult in cases when languages are not balanced, as the stronger language requires additional suppression. An important question therefore is whether switch costs depend on the direction of the switch, i.e., whether switching from a less dominant second language (L2) into the first language (L1) is more costly than vice versa. Depending on the modality in which the switch takes place, different findings have been reported.

In production tasks, numerous studies using various designs have reported asymmetric switch costs, where switches from L2 to L1 are more costly than switches in the other direction (Hernandez and Kohnert, 1999; Meuter and Allport, 1999; Costa and Santesteban, 2004; Philipp et al., 2007; Tarłowski et al., 2013). In contrast, in studies focused on language perception, such as during semantic categorisation or word recognition, similar costs were found for both switching directions (Thomas and Allport, 2000; Von Studnitz and Green, 2002; Macizo et al., 2012).

In language perception, the need for active selection of lexical items may not be present, and could therefore offer an explanation for observed symmetric switch costs (Macizo et al., 2012). Compared to production, language perception is mainly characterised by bottom-up processing and therefore language control in this modality is less dependent on endogenous control (Peeters et al., 2014). However, recent research outcomes demonstrated that asymmetrical switch costs can occur in a perception task (Pellikka et al., 2015), and similarly, production studies have not only reported asymmetric costs, but symmetric costs as well (e.g., Costa and Santesteban, 2004; Costa et al., 2006; Declerck and Philipp, 2015).

Several factors seem to modulate observed switch costs, among which preparation time, expectancy, language proficiency or language dominance. Longer preparation times or a more balanced language proficiency may reduce or eliminate switch costs, whereas language dominance is often linked to asymmetric costs (MacNamara et al., 1968; Verhoef et al., 2009; Declerck et al., 2013; Gullifer et al., 2013; Bultena et al., 2015; for a review, see Bobb and Wodniecka, 2013).

Event-related potential evidence on switch costs revealed modulations of the N400 effect in response to language switching. For example, in a sentence reading study, simultaneous interpreters showed an increased N400 effect after a switch from L1 to L2 (Proverbio et al., 2004a). The N400 effect was also observed for intrasentential language switches in L2 learners (Van der Meij et al., 2011). Also switches in the other direction, from L2 to L1, resulted in an N400-like effect (Ruigendijk et al., 2015; for a review on switch effects, see Van Hell and Witteman, 2009).

Early effects of language switching include the N1 component, a negative waveform peaking around 100 ms with the largest effects found at left anterior sites (Proverbio et al., 2004a). N1 peak amplitudes were modulated by expectedness and familiarity and indicate a state of lexical processing. Furthermore, the N1 component is possibly related to visual categorisation processes as well as the recognition of familiar letter strings (Proverbio et al., 2004b).

Another early language switching effect is the centrally distributed N250 component. In a masked priming study, the N250 appeared after a switch to L2, which was preceded by an unrelated masked L1 word (Chauncey et al., 2008). When the masked L1 word consisted of a direct translation equivalent of the following L2 word, a similar component with a slightly later peak at 300 ms was found as well (Midgley et al., 2009). The N250 component was also elicited after intrasentential language switches, although with a left-occipital distribution (Van der Meij et al., 2011). The N250 has been thought to reflect the ongoing processing of sublexical information, such as the mapping of specific letter combinations onto whole-word orthography (Holcomb and Grainger, 2006) for which language-specific changes might be detected (Van der Meij et al., 2011).

Further language switching effects include a large early left anterior negativity (a LAN-like response) over fronto-central electrode sites, elicited by code-switched words (Moreno et al., 2002). Integration efforts likely increased the memory load, as a result of attempting to integrate the syntactic structures of the languages under investigation. Moreno et al. (2002) further reported a late posterior complex (LPC) in the 450–850 ms time window, an effect often occurring when unexpected or improbable task-relevant items are processed. Participants with less experience in the switched language showed larger amplitudes, in line with such an interpretation. Similar LAN and LPC findings after language switches were also reported in a study with high proficient bilinguals (Ng et al., 2014). Additionally, also Van der Meij et al. (2011) reported an LPC in addition to other switching effects, which correlated with the language experience of their participants.

Language switches often elicit the N400, a component typically associated with semantic processing. Increased negative N400 amplitudes are elicited in response to semantic incongruities (Kutas and Hillyard, 1980 and their subsequent work). When comparisons were made between monolingual native speakers and bilinguals or L2 learners, the N400 proved to be sensitive to factors such as language proficiency and language exposure. For instance, compared to monolingual speakers, late bilinguals showed delayed peak latencies for the N400 even when they were tested in their first language (Ardal et al., 1990). Furthermore, the study showed that reduced N400 amplitudes were found for L2 stimuli. The authors argued that the N400 might therefore be sensitive to the automaticity of language processing. The finding of delayed N400 peak latencies in L2 learners was replicated by Weber-Fox and Neville (1996), although amplitudes of the N400 were similar to that of native speakers.

The ‘extended lexical search’ theory (Soares and Grosjean, 1984) attributes observed differences in N400 responses between L1 and L2 to the increased lexicon size in bilinguals. Bilingual speakers take more time to scan their lexicon in order to make sure that a particular word matches an entry in their lexicon. This therefore delays the onset of the N400. Alternatively, the absence of automatisation in L2 processing has been proposed to explain divergent N400 responses (Ardal et al., 1990).

Bilinguals and L2 learners differ from monolingual speakers in their language processing in various ways. Specific qualities of ERP components, such as the delayed and reduced N400, have often been found to mark differences between groups of monolingual, bilingual or second-language learners. According to psycholinguistic models, such as the Revised Hierarchical Model (RHM, Kroll and Stewart, 1994), L2 speakers move through certain stages in their L2 learning process. The RHM predicts that when an L2 learner starts learning their second language, the lexical items (lemmas) of that language are linked to their conceptual representations (meaning) through L1 items alone. In this stage, then, many direct translations to L1 are necessary to access the correct conceptual meaning of the L2 word. Later, as proficiency increases, the need for direct translation will decrease, as more direct links between L2 lemmas and the conceptual system have been formed along the way. A high proficient L2 speaker would therefore be able to directly match a conceptual meaning to an L2 word.

The RHM also offers a valid explanation for the reported differences between L2 speakers and native speakers regarding N400 responses to incongruent words. Delayed and/or reduced N400 responses would then reflect the extra time needed to reach the conceptual system or, alternatively, these deviant N400 responses reflect the insensitivity to the congruency of L2 words, because of weaker links to the conceptual system. A similar explanation was put forward by Van der Meij et al. (2011).

Language switching paradigms offer a unique viewpoint on this matter. According to Green (1998), a low L2 proficiency may result in a greater need to inhibit L1 during L2 use, especially during language production. Recent findings point to similar mechanisms in language perception (Pellikka et al., 2015).

The current study aims at investigating the impact of combining language switches with semantic incongruities in L2 learners with different language proficiencies. To this end, we used an experimental paradigm in which participants were presented with written sentences in L2, half of which contained a switch to L1. Fifty percent of the sentences furthermore included incongruent continuations. Two groups of L2 learners were tested; an intermediate proficiency (IP) and high proficiency (HP) group. To compare responses of L2 learners to a baseline, a control group of native speakers was tested as well.

We formulated several concrete predictions based on previous findings and theories: (1) Language switching will have a greater impact on less proficient L2 learners compared to advanced, highly proficient L2 learners, as more L1 inhibition is necessary when the L2 is not yet fully developed, in order to prevent language interference. This will likely be reflected in increased N250, N400 amplitudes and/or elicitation of LPC. (2) Semantic incongruities are easier to process in L1 than in L2, especially for L2 learners with lower proficiencies, as they rely more heavily on the links between L1 words and the conceptual system to assess meaning. We therefore expect that N400 responses to semantic incongruities are likely to yield larger amplitudes in L1 than in L2. Following prediction number 2, we could further predict that (3) Advanced L2 learners are more sensitive to semantic incongruities in L2 than L2 learners with a lower proficiency, as advanced learners have more direct access to the conceptual system via L2 words. Based on previous findings, we hypothesise that N400 amplitudes will be reduced and/or delayed in L2 learners with a lower proficiency, whereas highly proficient L2 learners may show responses similar to the control group of English native speakers. Our last prediction concerns the combination of language switches and semantic incongruities: (4) Due to the great amount of L1 inhibition during L2 language use, switches to L1 may interfere with L1 semantic processes needed to differentiate between congruent and incongruent words, and may therefore delay or inhibit these processes as well, characterised by decreased and/or delayed semantic N400 responses. Such L1 inhibition is expected to be greater in the low proficiency group, as L1 interference is more likely to occur here.

## Materials and Methods

### Participants

The control group of native English speakers consisted of 16 participants (7 female) aged 18 to 44 (mean age: 32.3 years). These participants grew up in a monolingual English-speaking environment during early childhood, although their current residency in Finland makes them L2 learners of Finnish as well [mean AoA 21.6 years, mean proficiency 2.6 on a scale from 1 (Elementary proficiency) to 5 (Native proficiency)].

To create two suitable L2 learner groups with an intermediate proficiency (IP) and high proficiency (HP) in their L2 (English), we recruited 59 potential participants, and administered an English aptitude test, the same test as used in Van der Meij et al. (2011). The test consisted of 60 multiple-choice questions concerning English grammar and vocabulary, and the final score was convertible into six levels, corresponding to the levels described by the Common European Framework of Reference for Languages (Council of Europe, 2001); 1 = A1 (Breakthrough), Level 2 = A2 (Waystage), Level 3 = B1 (Threshold), Level 4 = B2 (Vantage), Level 5 = C1 (Effective Operational Proficiency) and Level 6 = C2 (Mastery). Only individuals scoring Level 4 (CEFR B2 level, *n* = 12) and Level 6 (CEFR C2 level, *n* = 15) were invited as participants in the IP and the HP groups, respectively. Individuals scoring Level 5 were excluded to ensure a clear distinction between the two proficiency groups. The groups invited to take part in the experiment thus corresponded to two distinct proficiency levels, where the highly proficient group achieves the highest score possible for L2 learners, while the intermediately proficient group scores at the CEFR B2 level, which is sufficient to understand routine information, articles, reports and contemporary literary prose (ALTE; Association of Language Testers in Europe, 2016).

The remaining 27 (15 HP and 12 IP) participants in this group were all native speakers of Finnish. Regarding English as their L2, the IP group (mean age 31.2, 8 female) reported a mean age of acquisition (AoA) of 10.6 years and a mean language exposure of 20.6 years, whereas the HP group (mean age 32.0, 9 female) had a mean AoA of 8.6 years and language exposure of 23.4 years. Descriptive statistics on the L2 learners on various language background measures are presented in **Table 1**.

**Table 1:** Details on L2 measures for all L2 learners (HP; high proficient, IP; intermediately proficient), including their age (in years), their age of acquisition (in years), self-reported proficiency on a scale of 1–5, the score on the English aptitude test and L2 exposure (age minus AoA).

	Intermediate proficiency	High proficiency	*T*-test (*p*)
Men:Women	3:9	6:9	ns
Age	32.0 (5.1)	31.2 (6.3)	ns
L2 AoA	10.6 (2.5)	8.6 (2.1)	*p* < 0.05
L2 aptitude test score	37.1 (3.7)	53.7 (3.1)	*p* < 0.001
L2 self-reported proficiency	2.5 (0.5)	4.0 (0.0)	*p* < 0.001
L2 exposure	20.6 (6.4)	23.4 (4.1)	ns

*Mean values, standard deviations (SD) and *p*-values of *T*-tests between proficiency groups are reported.*

In order to investigate the effect of AoA on language proficiency, we performed a correlation analysis, with AoA and Test score as variables. The correlation between the two variables did not reach significance (*r*_τ_ = 0.24, *p* = 0.116, *N* = 27). L2 AoA and proficiency are often confounded and possibly here too, however, considering the absence of a correlation, proficiency remains the main factor of interest in this study.

All participants had normal hearing and normal or corrected-to-normal vision, showed no history of neurological damage, had no somatic or psychiatric conditions affecting cognitive functions (including major depression), no substance abuse, and did not take medication affecting cognitive functions. All participants signed an informed consent prior to the experiment and received 15–30 euros worth of cultural vouchers for their participation, depending on the length of the experiment. The study was approved by the Ethical Committee of the Faculty of Behavioural Sciences, University of Helsinki.

### Stimuli

The stimuli consisted of 276 sentences. Half of these were presented in English only (non-switched), the other half contained a switch to Finnish (switched). Each of the 276 sentences had four versions: it was semantically congruent or incongruent and contained a language switch or not. Only nouns were included as target words, in order to avoid controversial issues regarding the processing of nouns vs. verbs, especially because differences in the processing of verbs and nouns have been found after language switches (Ng et al., 2014).

Congruency was rated by 30 native speakers of English and 27 second-language learners of English (with various language backgrounds) by asking them to rate the likeliness of three different words to fit a sentence on a Likert scale from 1 (Not very likely) to 5 (Very likely). The final congruent target words all fell within the category ‘Very likely’ (*m* = 4.84 for English native speakers and *m* = 4.87 for L2 learners), whilst the incongruent target words were rated as ‘Not very likely’ or ‘Not likely at all’ (*m* = 1.54 for English native speakers, *m* = 1.59 for L2 learners). The decision to include incongruent target words rather than low cloze words, stems from earlier reported insensitivity to semantic subtleties by L2 speakers. The current design attempts to elicit large N400 responses, for which the use of incongruent target words makes a more probable fit.

Four different lists of the stimuli were created according to a Latin-square design, to ensure that each version of a sentence occurred only once in a list. For example, *Dan is polishing the shoe* did not occur in the same list as *Dan is polishing the sock* or *Dan is polishing kenkää/sukkaa (shoe/sock)*, to avoid familiarity of the stimuli. The number of congruent, incongruent and switched stimuli was kept equal throughout the lists, to ensure a balanced design. In this manner responses to 69 trials of each condition were obtained.

English native speakers were exclusively presented with 138 non-switched sentences. All non-switched sentences were 7–11 words long and had a similar Subject-Verb-Object structure. Each sentence started with a noun phrase or proper noun followed by a transitive verb in present progressive tense and a noun phrase in object position. Target words consisted of the head noun as direct object of the sentence. In 50% of the sentences, these target words formed a semantically congruent continuation of the sentence, and in the other half of the sentences they were semantically incongruent. The average Log frequency of the congruent words in the non-switched sentences was 2.00 with an average word length of 5.1 letters, and 1.96 and 5.0 for the incongruent words, respectively. To avoid sentence wrap-up effects (Hagoort, 2003), prepositional or adverbial phrases were added after the target word.

Finnish native speakers were presented with the same non-switched sentences, as well as switched versions of the sentences. The intrasentential language switches respect the grammatical structures of both languages and overlap them in an elegant way. This was done to avoid language switches that would not occur in real life (*natural code switches*, Poplack, 1980). Target words were mostly directly translated into Finnish, or replaced by equivalents if the directly translation did not fit the context or due to restrictions related to matching frequency and length. This resulted in an average Log frequency of 1.95 for the congruent Finnish words and an average word length of 6.9 letters. The incongruent Finnish words had an average Log frequency of 1.96 with an average word length of 6.7 letters. The frequencies were obtained from written word frequencies in the WordMill lexical database (Laine and Virtanen, 1999). Details about the experimental stimuli are presented in **Table 2**.

**Table 2:** Descriptive statistics on experimental variables, including log frequency, length and congruency ratings.

	English congruent	English incongruent	*T*-test (*p*)	Switched congruent	Switched incongruent	*T*-test (*p*)
Log frequency	2.00 (0.5)	1.96 (0.6)	ns	1.96 (0.5)	1.96 (0.7)	ns
Length	5.1 (1.5)	5.0 (1.3)	ns	6.9 (1.5)	6.7 (1.0)	ns
Congruency rating	4.86 (0.3)	1.56 (0.6)	<0.001	–	–	–

*Mean values, standard deviations (*SD*) and *p*-values of *T*-tests between variables are reported. Ns, non-significant.*

To ensure grammaticality of all sentences, Finnish words were inflected with either the partitive (to mark partialness of a noun) or genitive-accusative case (to mark totality of a noun).

For example, in its switched version, the word *room* in the sentence ‘The janitor is locking the room’ was inflected with the genitive-accusative case (*huoneen*) and not with the partitive case (*huonetta*), because the latter would yield an ungrammatical sentence, as a room cannot be partly locked. However, most of our experimental sentences contained the direct object in its partitive case, as the verb often dictates the use of this case, and there was no need to emphasise that something was done to the direct object *as a whole*. For example, we used ‘Dana syö leipä**ä**’ (Dana eats (some) bread) rather than ‘Dana syö leivä**n**’ (Dana eats the whole loaf of bread). Furthermore, often the use of the genitive-accusative case requires a change in the stem of the word (leipä → lei**v**än; consonant gradation) and we wanted to avoid these stem changes.

The grammaticality of all sentences was checked by a native speaker, who did not participate in the actual study. In the Finnish language, the definite article ‘the’ and indefinite article ‘a’ are not expressed, so in order to keep the sentences grammatical, the definite or indefinite articles preceding the target words were omitted. As Finnish is an agglutinative language, certain prepositions were added words as suffixes, instead of being expressed by separate words. To illustrate, the prepositional phrase ‘*in the morning*’ was translated as *aamulla*, consisting of the stem *aamu* + suffix -*lla* (addessive case). This resulted in differences in the total length of the sentences (total length varied from 5 to 7 words) compared to the non-switched sentences. However, these differences in sentence length occurred only after the target word, and are therefore assumed not to affect the experimental design. Examples of the experimental stimuli are found in **Table 3**.

**Table 3:** Examples of experimental stimuli. Incongruent words are starred with a ^∗^, language switches are displayed in bold.

Subject	Verb	Object – target word	Sentence ending
The boy	is tuning	*the guitar/the sun^∗^* ***kitaraa/aurinkoa****^∗^*	before the concert. ennen konserttia.
The woman	is watering	*the garden/the boy^∗^* ***puutarhaa/poikaa****^∗^*	inthe afternoon. iltapäivällä.

### Procedure

Each participant was pseudorandomly assigned to one of the four experimental lists. Within each list, there were two (non-switched stimuli only) or four blocks lasting about 15 min each. The stimulus presentation was commanded by a script written in Presentation 14.4 (Neurobehavioral Systems, Albany, CA, United States), which randomised the order of the sentences within each block. Prior to each sentence, the participants were shown a centred fixation cross that lasted for 800 ms. Thereafter, each word was presented separately for 400 ms with an inter-stimulus interval (ISI) of 500 ms.

The last word of each sentence was followed by an inter-trial interval (ITI) of 1000 ms, before the presentation of the next sentence. To ensure that the participants were paying close attention to the stimuli, 20% of the sentences were immediately followed by a question appearing on the screen, which they had to answer by pressing a button on a response box. The questions appeared at random intervals, and were simple yes/no-questions about the sentences they had just read. They either referred to the subject of the sentence, the main verb or the object that followed the verb. As the target words occurred only mid-sentence, possible motor interference is minimised.

Participants were instructed to read each sentence for comprehension and to sit as still as possible, avoiding head movements and excessive eye-blinking. Prior to the experiment, four practise trials were presented in order to familiarise the participant with the task. All participants were allowed to take a break in between the blocks, and were offered refreshments and snacks. The participants were rewarded with cultural vouchers worth 5 Euros per each half an hour. The experiment lasted approximately 60–90 min.

### Event-Related Potential Recording and Analysis

The electroencephalogram (EEG) was recorded with a sampling rate of 512 Hz and a recording bandwidth of DC-104 Hz with the BioSemi system (BioSemi B.V., Amsterdam, the Netherlands) by applying a 64-active-electrode cap. The CMS electrode (at the approximate location of PO1) was used as a recording reference. External electrodes were placed on the left and right mastoids and on the tip of the nose. An additional electrode was attached below the right eye to record the vertical electro-oculogram (VEOG). The participants were comfortably seated in a video- and audio-monitored, electrically shielded, and sound-attenuated chamber. Data analyses were performed with BESA Research 6.0 Software (BESA GmbH, Munich, Germany). The continuous EEG was offline filtered with a bandpass of 0.1–45 Hz. The data was offline re-referenced to the average of the mastoids. Any channels (maximum 10%) distorted because of technical malfunctioning were replaced by interpolating the data of the surrounding electrode sites (Perrin et al., 1989; Bendixen et al., 2008). An automatic eye-blink correction was performed on the data using a principal component analysis (PCA; Ille et al., 2002), and other remaining artefacts were removed automatically by using a ±100 μV rejection level. The EEG was epoched to a time period of 200 ms before and 900 ms after the stimulus onset, with a baseline correction of -200 to 0 ms.

Epochs were averaged separately for each condition. On average, 12% of the trials were excluded after artefact correction. The final data set included on average 61 congruent trials (*SD* = 7.3) and 58 incongruent (*SD* = 9.2) trials for the English native speakers. Recorded data of Finnish high proficient L2 speakers resulted in a final data set with an average of 61 (*SD* = 10.1) non-switched and 60 (*SD* = 10.6) switched congruent trials. For the Finnish intermediate proficient group this was 60 (*SD* = 10.0) and 62 (*SD* = 10.1), respectively. The incongruent stimulus trials included an average of 60 (*SD* = 10.0) and 61 trials (*SD* = 10.8), and switched incongruent versions 59 trials (*SD* = 11.4) and 61 trials (*SD* = 10.4), for the Finnish HP and IP group, respectively.

### Statistical Analysis

A 350–500 ms time window was chosen for the N400 component analysis after visual inspection of the waveforms. Mean amplitudes were calculated for each participant in each condition, using custom-made MATLAB (2012b, The MathWorks, Natick, MA, United States) scripts. Visual inspection of the scalp topography and waveforms showed that the N400 component was largest at mid-central and posterior areas, and nine midline electrodes, in which the signal was most prominent, were chosen for further statistical analysis: CP3, P3, PO3, CPz, Pz, POz, CP4, P4, PO4. For the non-switched dataset, a four-way mixed model ANOVA was conducted to test the effects of Congruency (two levels; congruent/incongruent), Hemisphere (HS, three levels: left, midline, right), and Anterior–Posterior division (AP, three levels: anterior, central, and posterior) on the mean amplitudes for the three groups (between-subjects factor Group: three levels).

To test modulation of the N400 during language switches, a separate four-way mixed model ANOVA was conducted to compare the effects of Congruency (two levels; congruent/incongruent), Switch (two levels; code switch/no code switch), Hemisphere (HS, three levels: left, midline, right) and Anterior–Posterior division (AP, three levels: anterior, central, posterior) on the mean amplitudes in the previously mentioned time window.

To further examine the difference between switched and non-switched stimuli, another four-way ANOVA was performed on a different set of nine electrodes, which best represented the distribution of the observed switch-specific effects: F5, FC5, C5, F3, FC3, C3, F1, FC1, C1. Effects of Congruency, Switch, AP and HS were tested in both proficiency groups during the time windows of 200–300 ms and 300–450 ms. A time window of 520–670 ms was further chosen to investigate late switching effects, on a set of nine right-lateralised parietal electrodes: CP2, P2, PO2, CP4, P4, PO4, CP6, P6, and PO8. To all mixed model analyses of variance, Greenhouse-Geisser corrections were applied wherever appropriate and *p*-values after correction are reported in the results. Bonferroni corrections were applied to *Post hoc* analyses.

## Results

### Semantic Incongruency in Non-Switched and Switched Stimuli (350–500 ms)

**FIGURE 1:**
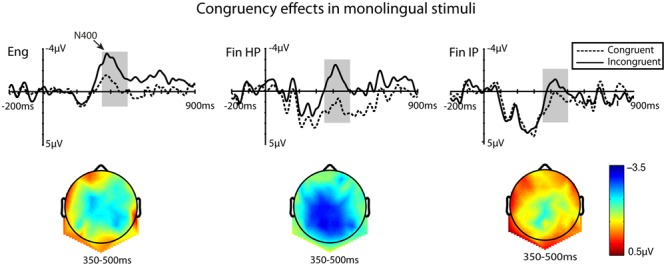
Grand-average waveforms taken from a region of interest (ROI) consisting of a set of nine centro-parietal electrodes. Each of the two non-switched conditions is plotted for the three groups: semantically congruent (dashed lines) and semantically incongruent (continuous lines). Accompanying scalp maps were obtained for the 350–500 ms time windows for the averaged values of difference waves (incongruent minus congruent). The *x*-axis represents time in milliseconds, and the *y*-axis represents amplitude in microvolts. The 200 ms baseline is plotted, and time 0 marks the onset of the target word.

The ANOVA (Congruency × HS × AP) on the mean amplitudes between 350 and 500 ms, showed a main effect of Congruency [*F*(1,41) = 27.64, *MSE* = 23.01, *p* = < 0.001, η^2^ = 0.409] and HS [*F*(2,82) = 20.16, *MSE* = 8.90, *p* = < 0.001, η^2^ = 0.335]. An interaction between Congruency and AP was also found [*F*(2,82) = 3.30, *MSE* = 3.07, *p* = 0.044, η^2^ = 0.076], demonstrating that the N400 was most prominent at central electrodes. **Figure 1** shows the region of interest (ROI) grand average time-locked ERPs for the three groups, including the scalp maps that show the distribution of the N400 effect over the scalp, after subtracting ERP activity elicited by congruent stimuli from the average activity elicited by incongruent continuations of the sentences.

**FIGURE 2:**
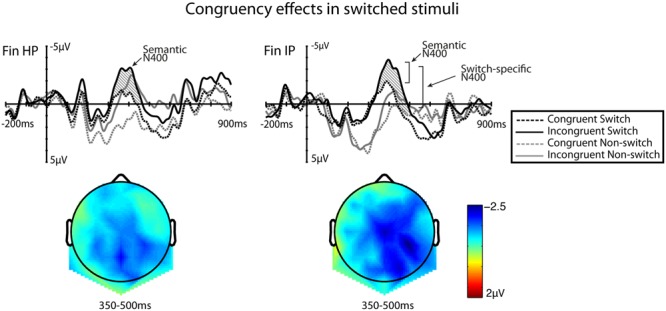
Grand-average waveforms taken from a ROI consisting of a set of nine centro-parietal electrodes. Each of the four conditions is plotted for the two L2 learner groups. The shaded region shows the semantic N400 effect that was found during switched items (the difference between congruent and incongruent words). Accompanying scalp maps were obtained for the 350–500 ms time windows for the averaged values of difference waves (language-switched incongruent minus language-switched congruent). The 200 ms baseline is plotted, and time 0 marks the onset of the target word.

For the semantic N400 effect during language switches, the ANOVA (Congruency × Switch × HS × AP) in the 350–500 ms time window showed a main effect of Congruency [*F*(1,26) = 23.14, *MSE* = 32.63, *p* = < 0.001, η^2^ = 0.481], Switch [*F*(1,26) = 5.54, *MSE* = 66.93, *p* = 0.027, η^2^ = 0.181], HS [*F*(2,52) = 19.77, *MSE* = 38.19, *p* = < 0.001, η^2^ = 0.442], AP [*F*(2,52) = 31.53, *MSE* = 32.87, *p* = < 0.001, η^2^ = 0.558] and a significant three-way interaction of Switch × HS × Group [*F*(2,52) = 4.26, *p* = 0.020, η^2^ = 0.146] as well as an interaction Switch × AP [*F*(2,52) = 6.46, *MSE* = 39.09, *p* = 0.007, η^2^ = 0.205]. **Figure 2** shows the ERPs that were elicited by the switched incongruent and congruent continuations of the sentences, compared to their non-switched versions. The accompanying scalp maps show the difference between language-switched incongruent items and language-switched congruent items.

**FIGURE 3:**
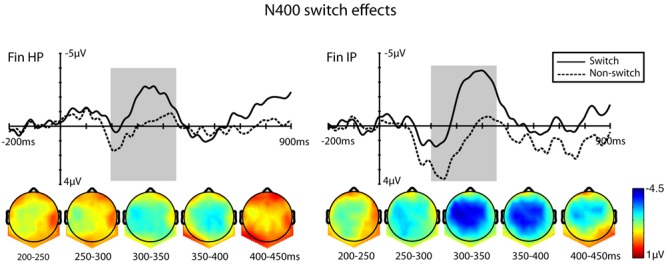
Grand-average waveforms taken from a ROI consisting of a set of nine fronto-central electrodes to represent the switching effects. Switched stimuli and non-switched stimuli are taken together and plotted for the two L2 groups. Accompanying scalp maps were obtained every consecutive 50 ms from 200 ms until 450 ms and reflect the averaged values of difference waves (language-switched minus non-switched). The 200 ms baseline is plotted, and time 0 marks the onset of the target word.

*Post hoc* analyses revealed that the switch effects were most clearly visible in the anterior region (mean difference anterior vs. central electrodes -1.243, *p* = < 0.001, mean difference anterior vs. posterior electrodes -2.036, *p* = < 0.001, η^2^ = 0.669). In the posterior parts, no differences between switches and non-switches were found. The three-way interaction Switch × HS × Group shows a left-lateralised response for switches in the IP group (mean difference switch vs. non-switch in left HS position 0.829, *p* = 0.037, η^2^ = 0.162, mean difference switch vs. non-switch in midline HS position 1.691, *p* = 0.049, η^2^ = 0.146), while the HP group did not show significant differences between switches and non-switches in any of the locations in this electrode set. These effects are illustrated most clearly in **Figure 3**. However, any clear interpretations of this interaction cannot be made, as the Switch effects for both groups are more pronounced in anterior scalp locations and analysed using a separate ROI, whereas the current ROI represents only part of the Switch effect.

### Early Language Switching Effects (200–300 and 300–450 ms)

In the early time window of 200–300 ms, the ANOVA (Congruency × Switch × HS × AP) showed a main effect of Switch [*F*(1,26) = 34.16, *MSE* = 22.12, *p* = < 0.001, η^2^ = 0.577] and HS [*F*(2,52) = 4.01, *MSE* = 10.46, *p* = 0.048, η^2^ = 0.138], and furthermore an interaction of Switch × AP [*F*(2,52) = 5.59, *MSE* = 0.92, *p* = 0.014, η^2^ = 0.183], for which switches yielded the largest negative amplitudes at fronto-central electrodes. **Figure 3** includes a plot of ROI ERPs for switched and non-switched stimuli, and scalp maps in 50 ms consecutive time windows that show the similarity of the topographical distribution of the negativity after subtracting ERP activity of non-switched items from their language-switched versions.

In the 300–450 ms time window, the ANOVA (Congruency × Switch × HS × AP) showed a main effect of Congruency [*F*(1,26) = 10.40, *MSE* = 26.04, *p* = 0.003, η^2^ = 0.294], Switch [*F*(1,26) = 37.83, *MSE* = 39.11, *p* = < 0.001, η^2^ = 0.602], and HS [*F*(2,52) = 8.78, *MSE* = 11.87, *p* = 0.002, η^2^ = 0.260]. A two-way interaction Congruency^∗^HS [*F*(2,52) = 8.07, *MSE* = 0.68, *p* = 0.003, η^2^ = 0.244] showed significant congruency effects in all hemisphere positions, but particularly large negative responses in the right hemispheric parietal electrodes after presentation of semantically incongruent stimuli (mean difference between amplitudes for left HS vs. midline HS position 0.716, *p* = 0.009, mean difference for midline HS vs. right HS position 0.455, *p* = 0.050 and mean difference left HS vs. right HS position 1.171, *p* = 0.003), whereas differences between HS position were not found for congruent stimuli.

Furthermore, we found a two-way interaction of Switch^∗^AP [*F*(2,52) = 5.93, *p* = 0.009, η^2^ = 0.192], indicating that, although the switch effects was seen in all positions, differences in amplitudes between the electrodes along the three anterior–posterior positions were found only during switches, and the most negative values were found for the central electrodes (mean values during switch for anterior electrodes -2.334, central electrodes -2.858 and posterior electrodes -2.589, mean difference between amplitudes for anterior electrodes vs. central electrodes 0.524, *p* = 0.010, mean difference for central electrodes vs. posterior electrodes 0.269 *ns, p* = 0.288 and mean difference anterior electrodes vs. posterior electrodes 1.171 *ns, p* = 0.804).

### Late Switching Effects (520–670 ms)

**FIGURE 4:**
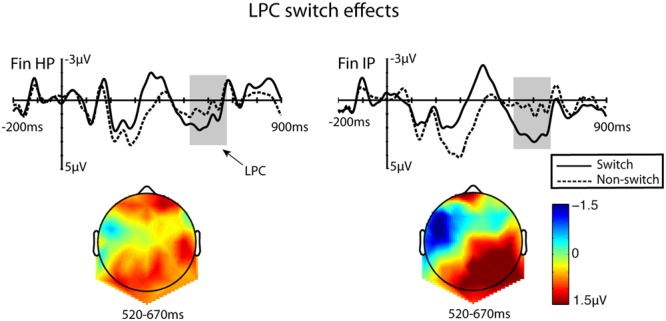
Grand-average waveforms taken from a ROI consisting of a set of nine right-lateralised parietal electrodes to represent the late positivity found after language switches. Accompanying scalp maps were obtained for the 520–670 ms time windows for the averaged values of difference waves (language-switched minus non-switched). The 200 ms baseline is plotted, and time 0 marks the onset of the target word.

In this later time window, the ANOVA (Congruency × Switch × HS × AP) showed a main effect of Congruency [*F*(1,26) = 4.78, *MSE* = 41.13, *p* = 0.038, η^2^ = 0.161], indicating a residual activity of the semantic N400 effect, with more negative values for the incongruent condition compared to congruent items, and Switch [*F*(1,26) = 4.34, *MSE* = 101.32, *p* = 0.048, η^2^ = 0.148], where switches elicited significantly more positive amplitudes than non-switches. A main effect was also found for AP position [*F*(1,26) = 6.88, *MSE* = 9.57, *p* = 0.005, η^2^ = 0.216]. In **Figure 4** the ROI ERP waveforms are plotted, and accompanied by scalp maps that reflect the topographical distribution of the late positivity in this time window, obtained by subtracting responses to non-switched stimuli from ERP activity after language switches. These results confirm the late posterior positivity complex (LPC) in reaction to language switches, with more positive mean amplitudes for switches in this time window compared to non-switches (1.886 vs. 0.533, respectively), although no significant interaction between Group and Switch was found. The most positive amplitudes were found in parietal electrodes (mean amplitudes for anterior electrodes 0.756, central electrodes 1.514 and posterior electrodes 1.357).

### Summary of Results

In the time window of 350–500 ms, semantic incongruency elicited a large broad centro-parietally distributed N400 component in the omnibus ANOVA that included all three groups (Native speakers of English and intermediate and high proficient learners of English). Both L2 learner groups were exposed to switches to Finnish, and despite the language switch, incongruent and congruent target words elicited a centro-parietally distributed semantic N400 effect.

Language switches revealed an early negativity in the 200–300 ms time window in both groups, characterised by a fronto-central distribution, accumulating in a large N400-like negativity in the 300–450 ms time window. In the 520–670 ms time window, language switches elicited a posterior positivity. This posterior complex (LPC) was distributed over parietal areas of the scalp.

## Discussion

Here, we investigated the interplay between semantic processing, language switching and language proficiency. More specifically, we examined cortical correlates of lexical processing during language switching. Data were obtained from a control group of English native speakers reading English sentences, of which half contained semantically incongruent words in the object position of the sentence, whereas the other half of the sentences was semantically congruent. The Finnish native speakers (L2 learners of English) were additionally exposed to sentences that contained intrasentential switches to Finnish (L1), in semantically congruent and semantically incongruent contexts. The effect of language proficiency was further investigated by dividing L2 learners into two groups: intermediate proficiency (IP) and high proficiency (HP) L2 learners.

Our results on the N400 effect in monolingual English sentences did not show significant group differences between native speakers of English, IP and HP L2 learners, even if the effect seemed visibly smaller in the IP group. Thus, for the L2 learner groups that were tested in the current study, proficiency did not play a crucial role in semantic processing. These outcomes demonstrate that native-like levels of semantic processing may appear after considerable language experience, already when intermediate language proficiency levels are reached. This reasoning supports the convergence hypothesis by Green (2003), stating that non-native speakers may process and represent their L2 in the same way as native speakers of that language, as soon as their L2 proficiency improves. An increasing amount of studies focusing on semantic processing have brought forward evidence that supports the convergence hypothesis (Consonni et al., 2013; and for reviews, see Indefrey, 2006; Abutalebi and Green, 2007; Abutalebi, 2008).

Consequently, the current study did not replicate earlier findings that L2 learners with lower proficiency levels show deviant N400 responses (e.g., Ardal et al., 1990; Hahne and Friederici, 2001). One of the reasons why we did not find a difference in the size of the semantic N400 is probably because the L2 learners that we tested, were characterised by higher proficiencies on average compared to previous studies. The average English language proficiency level in Finland is very high, ranking #5 in a comparison of 72 countries (Education First English Proficiency Index). In light of the Revised Hierarchical Model (RHM, Kroll and Stewart, 1994), this implies that the IP group has already been able to form adequate links between L2 lexical entries and their conceptual representations, which are automatic and efficient enough to integrate the unfolding semantic information contained in sentences. Mediation through L1 lemmas was therefore not needed to process this semantic information. This finding is corroborated by a recent study on L2 comprehension, which showed that less proficient late L2 learners are able to access the meaning of an L2 word without activation of its L1 translation equivalent. The L1 word is only activated after the meaning of the L2 word has been accessed (Guo et al., 2012; Ma et al., 2017).

Furthermore, one of our main findings contradicted a prediction based on outcomes from previous studies and theories on language switching and trajectories of L2 learning. We hypothesised that a language switch to L1 may interfere with semantic processes at play when attempting to integrate target words into their sentence context, due to active L1 inhibition during L2 use. However, the current results did not confirm this hypothesis. Language switches elicited an N400-like response with a fronto-central distribution in the 300–450 ms time window. Manipulation of semantic congruency in these switched words further elicited an additional, centro-parietally distributed semantic N400 effect in both L2 learner groups, in the 350–500 ms time window. Contrary to our expectations, language switching did not interfere with ongoing semantic processes, as the participants still showed sensitivity to manipulations of congruency, i.e., between words that fit the sentence context, and words that did not. The absence of impeded semantic processing could be taken as evidence for non-selective access, contributing to a growing body of research on this topic (e.g., Dijkstra and Van Heuven, 2002; Thierry and Wu, 2007). The unhindered access to L1 items here indeed suggests that these L1 items are available for integration into the preceding sentence context, even if that context was presented in L2.

Other switch-specific effects in this study included an early switching effect around 200 ms, characterised by a large fronto-central negativity which continued and overlapped with the earlier described N400-like effect in the 300–450 ms time window. A similar early effect in response to language switches was first described in Moreno et al. (2002) as a LAN-like effect. The authors hypothesised that this response could indicate an attempt to integrate the syntactic structure of both languages into a coherent whole, thereby heavily relying on working memory. Other studies found early switching effects in the form of an N250 component and attributed these to an increased effort to integrate sublexical information, such as clusters of letters, with whole-word representations (Chauncey et al., 2008, see also Grainger and Holcomb, 2010). An alternative explanation of the N250 component relates to the detection or activation of orthographic or phonological rules specific to the other language (Van der Meij et al., 2011). It is plausible that the early negativity observed here in the 200–300 ms time window is related to the additional working memory load caused by the activation of two lexicons at the same time, or alternatively, due to the integration of the syntactic structures of both languages. This integration might aid the build-up of the semantic structure of the sentence, and the presence of a semantic N400 effect in both learner groups contributes to the view that such a process has been successful.

In previous studies, the N400 component observed after language switches has been suggested to reflect a greater effort in the integration of lexical and semantic representations (e.g., Chauncey et al., 2008) or the effort that is necessary to overcome the inhibition of the previous language (e.g., Pellikka et al., 2015). As pointed out above, however, our results show that this possible language inhibition does not necessarily hinder or limit semantic processing. Instead, processes related to semantic integration may occur in parallel with possible executive control processes. A similar account was also brought forward by Moreno et al. (2002), who proposed that at the semantic level, language switches occurring in sentence context are not necessarily more difficult to process than non-switched words. Instead, language switches might be processed as unexpected events, and therefore observed costs could arise from a more general non-linguistic level, such as the competition of task schemas. In the current study, the observation of distinct components after language switches and semantic incongruency indeed suggest at least partially different neural mechanisms underlying these processes.

Thus, early switching effects seen in the 200–300 ms time window may reflect an increased working memory load, while later switching effects in the 300–450 ms time window are likely due to the unexpectedness of the event. Our further results showed that language switches elicited an LPC in the 520–670 ms time window. This late positivity can also be interpreted as a reflection of the switch being an unexpected event, and a similar interpretation was provided by Moreno et al. (2002) and Van der Meij et al. (2011). These studies suggested that language proficiency may affect the perception of language switches (i.e., how unexpected they are), and could be due to the automaticity of language processing. In the current study, however, this explanation does not apply, because no interaction was found between proficiency and LPC effect, meaning that intermediate and high proficient L2 learners did not show significant differences in the size of the LPC. Again, this might be due to the relatively high proficiency in English even in our intermediately proficient L2 learner group. This group scored at the CEFR B2 level, whereas the low proficiency L2 learners in Van der Meij et al. (2011) scored on the CEFR A2 level, which is significantly lower. The bilinguals in the study by Moreno et al. (2002) reported near-native fluency and frequent use of both of their languages in non-classroom situations, and although detailed information about those bilinguals was not available, this suggests that they might have been early bilinguals. Direct comparisons between outcomes of these studies and the current one, are therefore challenging to make. Moreover, the LPC was elicited both after congruent and incongruent switches to L1. This outcome was also found in a study on auditory comprehension in high-proficient, late bilingual learners (FitzPatrick and Indefrey, 2014).

## Conclusion

The present study investigated the interplay between language switching and semantic processing, in a typical N400 semantic congruency design supplemented with switches to L1. To the best of our knowledge, this is the first study to combine these aspects in order to scrutinise the effect of language switching on semantic processes in different proficiency groups. We showed that individuals with an intermediate and a high proficiency in their L2 are sensitive to congruency differences between L2 words, demonstrated by semantic N400 effects that are similar to native speakers. Furthermore, our results suggest that switches to L1 resulted in a cost as measured by an N400, but did not impede semantic processing of the target words. Thus, we propose that language switching is not necessarily costly on the semantic level, but semantic processing can occur in parallel with executive control processes related to task switching.

Our findings are an important addition to the debate on language switching costs, as an increasing number of studies report no costs in response to language switches. Here, we show that language switching induces costs, while ongoing semantic processes remain unhindered. This should be considered in neurocognitive language inhibition theories and models of L2 language processing. The adaptive language control hypothesis by Green and Abutalebi (2013), also considers in more detail the impact of interactional context of language use, and how the language control network adapts to these constantly changing settings. Even though L1 inhibition may take place during the use of L2, quick bottom-up lexical access is possible this may especially help L2 learners when they attempt to unravel the semantic content of L2 sentences. By allowing quick activation of (perhaps even pre-activated) L1 equivalents, comprehension of the linguistic input can be maximized. This is also helpful in case they encounter L2 words that are unknown, but which meaning can be predicted. Possibly, in language production, the flexible use of both languages might even provide additional advantages, but it is beyond the scope of this article to discuss this in depth. Still, it would be interesting to test whether unhindered L1 access would benefit L2 learners with a proficiency that is lower than tested here. Psycholinguistics models such as the BIA+ (Van Heuven and Dijkstra, 2010) allow such language non-selective access, and account for switch costs through language task schema inhibition, rather than inhibition at the level of the language node. The language task schema may be altered depending on the situational context as well. This allows second-language learners to flexibly use their linguistic knowledge, so that ultimately, functional linguistic exchange can take place.

## Ethics Statement

This study was carried out in accordance with the recommendations of the Ethical principles of research in the Humanities and Social and Behavioral Sciences; formulated by the University of Helsinki Ethical review board in Humanities and Social and Behavioral Sciences. All subjects gave written informed consent in accordance with the Declaration of Helsinki. The protocol was approved by the University of Helsinki Ethical review board in Humanities and Social and Behavioral Sciences.

## Author Contributions

SH has been responsible for the conception and design of the work, the acquisition of data, data analysis, and data interpretation. She has furthermore drafted the work, approved of publication of this work’s version and agrees to be accountable for all aspects of the work in ensuring that questions related to the accuracy or integrity of any part of the work are appropriately investigated and resolved. AL has been responsible for guiding the conception and design of the work, and data analysis. She guided in data interpretation and has furthermore revised the work, approved of publication of this work’s version. She agrees to be accountable for all aspects of the work in ensuring that questions related to the accuracy or integrity of any part of the work are appropriately investigated and resolved.

## Conflict of Interest Statement

The authors declare that the research was conducted in the absence of any commercial or financial relationships that could be construed as a potential conflict of interest.
